# Modal Analysis Using Digital Image Correlation Technique

**DOI:** 10.3390/ma15165658

**Published:** 2022-08-17

**Authors:** Peter Frankovský, Ingrid Delyová, Peter Sivák, Jozef Bocko, Jozef Živčák, Michal Kicko

**Affiliations:** Faculty of Mechanical Engineering, Technical University of Košice, Letná 9, 042 00 Košice, Slovakia

**Keywords:** digital image correlation (DIC), experimental modal analysis, operational modal analysis, frequency response function

## Abstract

The present paper discusses a new approach for the experimental determination of modal parameters (resonant frequencies, modal shapes and damping coefficients) based on measured displacement values, using the non-contact optical method of digital image correlation (DIC). The output is a newly developed application module that, based on a three-dimensional displacement matrix from the experimental measurement results, can construct a frequency response function (FRF) for the purpose of experimental and operational modal analysis. From this frequency response function, the modal parameters of interest are able to be determined. The application module has been designed for practical use in Scilab 6.1.0, and its code interfaces directly with the ISTRA4D high-speed camera software. The module was built on measurements of a steel plate excited by an impact hammer to simulate experimental modal analysis. Verification of the correctness of the computational algorithm or the obtained modal parameters of the excited sheet metal plate was performed by simulation in the numerical software Abaqus, whose modal shapes and resonant frequencies showed high agreement with the results of the newly developed application.

## 1. Introduction

Non-contact optical methods with the possibility of all-field analyses are among the current trends in the field of experimental mechanics. Laser Doppler vibrometry (LDV), electronic speckle interferometry, digital speckle shearography and digital image correlation fall into this field [1]. In vibration analyses, laser vibrometry is a frequently used method for practical reasons. The advantages of the laser vibrometry method are high sensitivity, frequency range, high accuracy, measurement speed and the ability to measure over large distances. The main disadvantage is the high cost of the measuring equipment. LDVs have a wide range of applications in science and research, as evidenced by the number of published papers devoted to experimental modal analysis, operational modal analysis, operational vibration waveform analysis, and vibrodiagnostics. Šároši et al. [2] used the LDV method for a comprehensive analysis of the blade during its rotation. Specifically, they dealt with experimental modal analysis of the disc in a stationary state, oscillation shape analysis at operating speed—5000 rpm, and run-up analysis, which aimed to investigate the effect of the rotation speed on the change of the natural frequencies of the disc. A laser Doppler vibrometer and an optical derotator were used as measuring equipment. Zucca et al. [3] used the LDV technique to determine the frequency response functions and operating responses of 24 turbine blades at a specific angular velocity. Stanbridge et al. [4] streamlined the sensing approach which allowed modal shapes to be obtained from a relatively small set of measured data. Trebuňa et al. [5] used a laser vibrometer to identify the source of excessive vibration of a gas compressor discharge line. The use of a laser vibrometer was necessary in this case as the vibration level limited the use of accelerometers. The operational modal analysis is also addressed using LDV in the following papers [6,7].

Digital image correlation (DIC) is another method that has potential for use in vibration analysis [8]. However, for these purposes, a measurement system with high-speed cameras must be used. Although the DIC method is primarily used for component testing and determination of material properties [9,10,11,12], various publications report its use for vibration analysis and experimental modal analysis as well. There are also known publications where DIC is used for in-plane or in-space motion analysis [13,14].

The principle of the DIC method was described in the 1980s [15,16], but it did not take off until the late 1990s when advances were made in the fields of computing and digital optics. The method allows for the observation of various phenomena during deformation and motion of an object, which may be composed of a wide range of materials. The basis of DIC consists of scanning an analyzed surface onto which a randomly arranged speckle pattern has been applied, for example, by spraying black paint onto a white substrate. It is also common practice to print a stochastic pattern onto a white vinyl film [17,18].

The scanned area is divided into a number of elements called facets, which are chosen so that each of them contains contrasting black and white patches. The random pattern makes each of the facets unique. The resulting relative displacements are determined by correlating (comparing) the respective facets in the pre- and post-deformation states, or with respect to some reference step. Tracking a set of points on the surface of an object using a dual camera system is identical to the principle of creating a 3D image with human vision, i.e., the resulting displacement fields can be obtained in three directional axes (X, Y, Z) [1]. The principle of the DIC method is described in detail in [19].

Since the DIC method measures responses in the form of displacements, the frequency response functions are in the form of receptive fields. The sensitivity of the measurement system depends on the resolution of the CMOS sensors and the size of the area to be analysed. The frequency range is limited by the sampling rate of the cameras, i.e., the reciprocal of the minimum shutter time. High-speed cameras with sampling rates up to several hundred thousand frames per second (fps) at full sensor resolution are also currently in use. If the area of interest is reduced, the fps will increase. For measurements with high frame rates, additional illumination is necessary to ensure optimum lighting conditions. For this purpose, high-power reflectors with achromatic light are commonly used. The main advantage of DIC is the ability to capture responses from all points of the area under investigation at the same time and under the same excitation conditions. This saves time compared to commonly used methods as it eliminates the need for repeated pre-positioning of sensors over the surface of the area to be sensed. The disadvantage of this method is the time cost associated with correlation. The density of the grid of points and the accuracy of the measurement are dependent on the size of the selected facets. In [20], the authors investigated the effect of the size of the facets on the accuracy of modal parameter determination using the DIC method. They found that the size of the facet does not affect the natural frequencies, but if the chosen facets are too small, correlation errors occur which can cause inaccuracies in the determination of the frequency response functions. The size of the facets has a significant effect on determining the absolute amplitudes of the modal shapes. Depending on the size of the facets, the results can vary by up to several tens of percent.

The DIC method is not very often used for vibration analysis, but there are several publications whose authors have used this approach. While some of them report on the measurement of operational vibration waveforms or harmonic vibration analysis, there are also some that address the problem of experimental modal analysis and operational modal analysis, respectively. However, these approaches require a dditional numerical processing of the measured data and are more time consuming [21,22,23,24,25]. For the experimental modal analysis problem, it is also necessary to measure the excitation signal.

Ha et al. [21] described a method that determines the natural frequencies and the corresponding modal waveforms based on the processing of the data measured by the DIC method. They used an artificial beetle wing as the sensing object, which was excited by a random signal. To eliminate the influence of noise in the measurement, they used a spectrum averaging technique and Savitzky-Golay filter. The results achieved by the study were verified with numerical computation, which showed an identical solution. Wang [22] used high-speed DIC to determine the modal parameters of a composite panel reinforced with an aluminum L-section beam. In his work, Reu [23] comprehensively compared the LDV and DIC method for the purpose of all-field analysis of vibration and modes of vibration. He carried out response measurements on a flat steel plate, deposited at its corners, built up by a pseudo-random signal using a shaker. In the paper, he defined the advantages and disadvantages of the two techniques. Ehrhardt et al. [24] dealt with a similar study where they compared the modal shapes and corresponding natural frequencies of a planar steel beam embedded at both ends and a rectangular aluminum plate embedded around the perimeter using LDV and DIC. They found that both methods show comparable results in several aspects; however, the DIC method is more accurate in measuring more complex spatial deformations with higher amplitudes, whereas the LDV method is more sensitive at lower amplitudes and smaller deformations. Trebuňa et al. [25], using the DIC method, solved an operational modal analysis on a steel fan blade that was acoustically excited by white noise. They used the FDD (Frequency Domain Decomposition) method to determine the modal parameters from the resulting PSD (Power Spectral Density) matrix.

## 2. Digital Image Correlation Method (DIC)

The Digital Image Correlation method is one of the experimental optical mechanics methods. It is one of the non-contact optical methods capable of measuring deformations and displacements over the entire surface of the object under analysis. It is used for measuring all basic types of stresses—tension (compression), shear, torsion, and bending, as well as for combined stresses in the static and dynamic domains. DIC can be applied to measure very small (micro) areas as well as large areas, and the measurement results can be easily verified by FEA or strain gauge methods.

**Figure 1:**
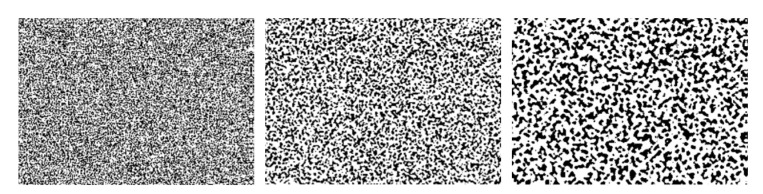
Stochastic patterns of different speckle.

The principle of the DIC method is the comparison (correlation) of the sensed stochastic (random) pattern (Figure 1), which is applied on the surface of the measured object during loading [26]. The sensed images are recorded using digital cameras, and the applied pattern reproduces the behaviour of the surface of the loaded object, with which it deforms simultaneously.

Depending on the dimensions of the analyzed object, the size of the spots of the random pattern is chosen. There are a number of application approaches. For example, for patterns of size 50 μm and larger, the spray gun method of spraying the contrast dye is used. Patterns of 50–15 μm are applied using Xerox toner powder to a wet white surface. Patterns of larger speckles are applied, for example, with a brush or roller with projections dipped in the contrast colour relative to the surface to be measured. Another method is to print a pattern ranging from 0.25 mm to several mm on vinyl film. To obtain good quality measurement results, a strong colour contrast of the pattern is necessary, ideally a combination of black and white matte colours without glare [19,27,28].

**Figure 2:**
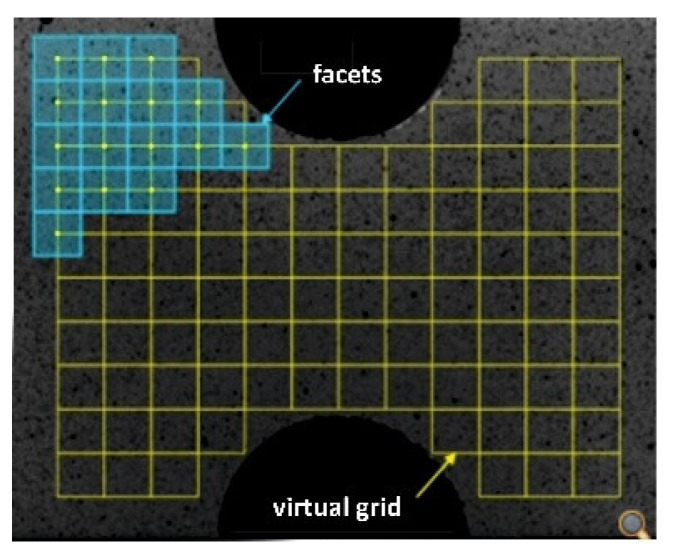
Facets with virtual grid.

Image correlation consists in dividing the area of the measured region into a finite number of geometric elements called facets (Figure 2). These are unique due to the random pattern, but it is necessary to adapt the pattern so that each of the facets contains contrasting black and white patches.

**Figure 3:**
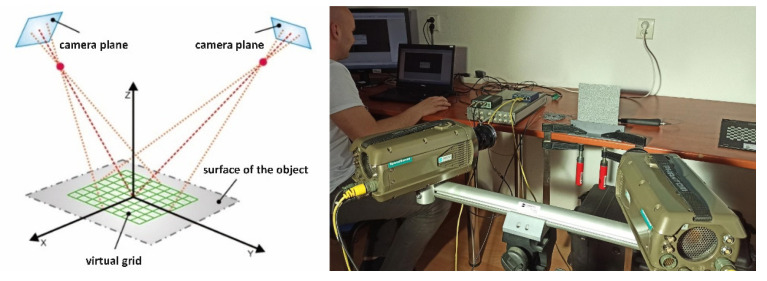
Q-450 measuring system with stereoscopic Phantom v310 camera arrangement.

The behaviour of each facet is characterized by its center point, thus by comparing the facets before and after loading, information about the displacements and deformations of all points on the surface under analysis can be obtained [28,29,30,31,32]. For the purpose of the problem addressed in this work, the spatial image correlation method was used, which employs the principle of image acquisition using a multi-camera system with a stereoscopic camera arrangement (Figure 3) [28]. If the relative position of the cameras, the magnification of the lenses, and the internal and external calibration values are known, it is possible to obtain the three-dimensional parameters of each point and thus construct a spatial contour. For our analysis, a 1-megapixel Phantom v310 high-speed digital camera (Figure 3) was used, which has a dedicated CMOS sensor with a resolution of 1280 × 800. At full resolution it allows 3250 fps, and at lower resolutions up to 500,000 fps.

## 3. Application Module for Modal Analysis

The ISTRA4D software is capable of recording and evaluating measurement data using the DIC optical method. It offers various options for the needs of laboratories and industrial practices. It allows further extensions in the form of post-process applications. These applications, also called application modules, are programmed in Scilab software (a freeware version of Matlab) and interfaced with ISTRA4D software, where the modules transform the measurement results according to defined mathematical functions.

In this paper, the authors discuss the development and description of an application module for the determination of modal parameters based on experimental modal analysis (EMA) and operational modal analysis (OMA) from data obtained by digital image correlation (DIC) measurements [28].

**Figure 4:**
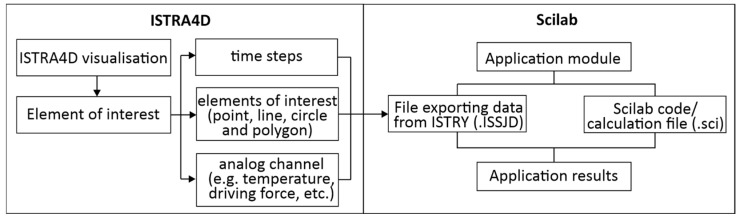
ISTRA4D—Scilab relation.

The relationship between the ISTRA4D software and Scilab is described in Figure 4. Based on the visual representation of the measured object, it is possible to identify a point, curve or polygon on its surface that defines our region of interest on the analyzed object. From the ISTRA4D software, it is then possible to export displacements, relative deformations, point coordinates, and time characteristics. It is also possible to obtain the outputs of the analog channel, which represents, for example, the excitation signal. The export of this data is then processed by Scilab.

The application module consists of two main files. They are text documents in the format:

- .ISSJD, which defines the export of the required ISTRA4D measurement data to Scilab.
- .SCE or .SCI, which works with the exported measurement data and transforms them on the basis of mathematical-physical relations into the required resulting parameters. It is the script itself created in Scilab.

The first interface of the modal analysis application module focuses on EMA, i.e., the estimation of modal parameters based on data from the frequency response function (FRF), which is influenced by the excitation frequency spectrum [28].

## 4. Measurement of Input Data Using DIC for EMA Solution

The application module was built based on a series of real sample measurements using the DIC method. For this purpose, a simple rectangular steel plate was chosen as the analyzed object, which had an analyzed area with the dimensions 156 × 152 mm and a thickness of 0.8 mm. The analyzed sample was made of DC03 steel. It is a deep-drawn steel, suitable for automotive body interior parts and other mouldings.

**Figure 5:**
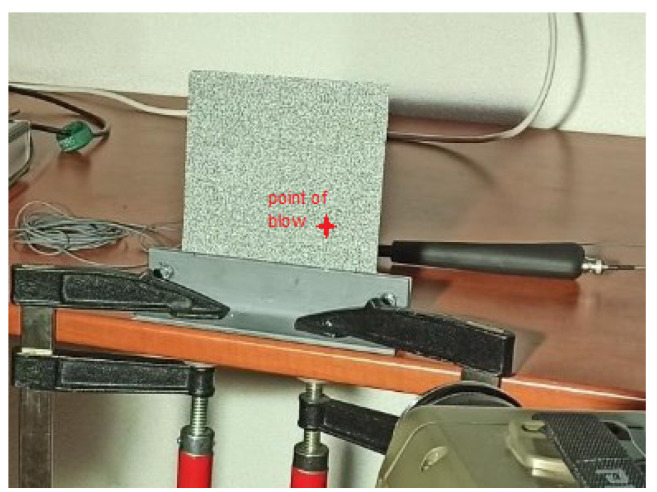
Laying of the analyzed steel plate and the excitation point.

The stochastic pattern was applied to the analyzed area in the form of pre-printed spots on a vinyl film. The steel sheet was woven at the bottom edge using two L-sections connected by screws (Figure 5). Figure 5 also shows the excitation location (EMA) where the impact hammer blow was located on the back side of the steel sheet [28].

The measurement was carried out with the Q-450 system from Dantec Dynamics using high-speed cameras. Calibration and correlation of the images were carried out using Istra4D software [26,28].

The measurement and evaluation conditions were as follows:

- Image acquisition time = 1 s;
- Frame rate = 5000 fps;
- Facet size = 21 pixels;
- Application grid spacing = 17 pixels.

**Figure 6:**
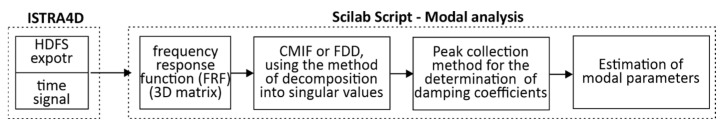
Procedure for processing measured and exported data.

The processing procedure of the measured and exported data for the determination of modal shapes and corresponding natural frequencies by experimental modal analysis is shown in Figure 6.

**Figure 7:**
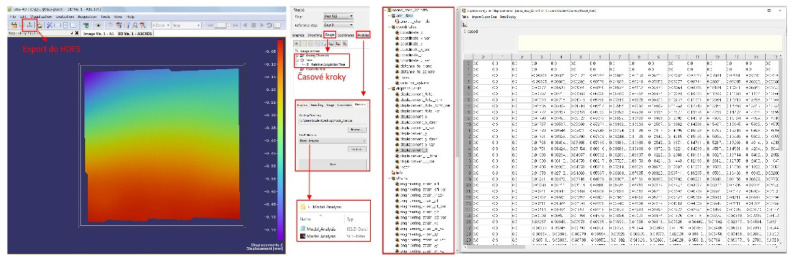
ISTRA 4D working environment and .HDF5 file matrices for time step 82.

In the first step of the solution, all measurement images were exported from the ISTRA4D software into hierarchically arranged. HDF5 files containing temporal information, frame counts and displacement matrices (Figure 7) [33]. The images were exported in the form of HDF5 files, with 2004 frames out of 5000 exported in total to reduce the computation time required. For the needs of the application module we developed, only the displacement matrices in the directions of all three axes are sufficient. The displacement matrices are loaded directly by a command from Scilab (.SCE file), which operates on the data of one of them as needed [28].

**Figure 8:**
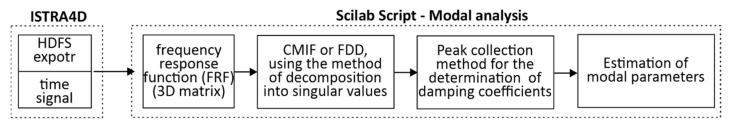
Processing of exported measurement data in Scilab.

The methodology for processing the measured and exported data using the Scilab mathematic-physical relationships is defined in Figure 8. The .SCE file, which represents a computational script written in the Scilab programming language, must be placed in the same folder as the .ISSJD file that defines the export of the time increments.

**Figure 9:**
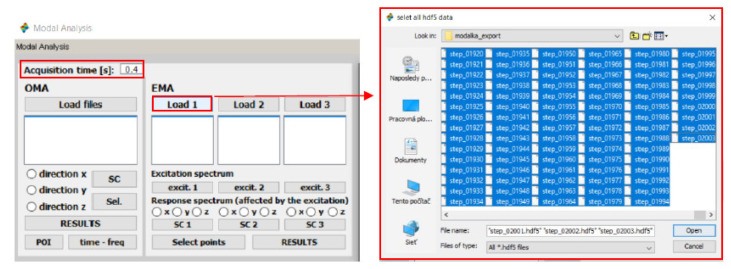
Initial GUI window for determining modal parameters.

Starting the application displays a window (Figure 9) that automatically defines the acquisition time based on the number of selected .HDF5 files.

For the demonstration of the application module, only one measurement file was used, i.e., the system was built by an impact hammer strike at only one location; hence, we will only look for one singular curve.

The ISTRA4D software stores the temporal data from the measurements in terms of a column vector and orientates the spatial data into row vectors. Scilab command lines define the input parameter time, which is automatically calculated as the difference between the second and first time steps multiplied by the number of selected .HDF5 files. Since the acquisition time was 1 s at 5000 fps, when considering 2000 frames, the time is adjusted to 0.4 s.

The first function requires the user to select the path to the exported .HDF5 files from the measurement to determine the number of time steps and the naming of the files to work with next. The quantities that are also required between functions are always defined as a global parameter.

**Figure 10:**
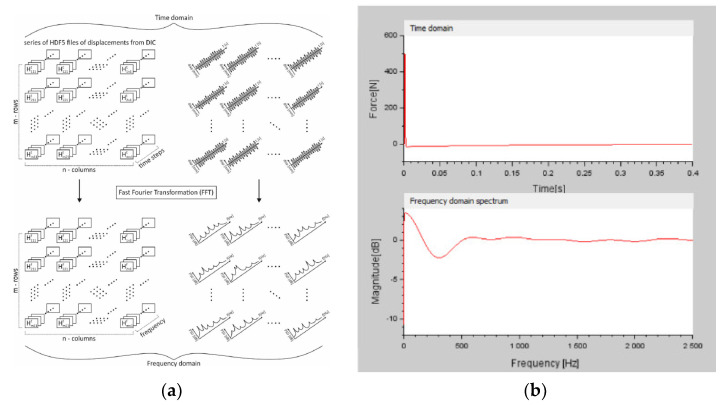
(**a**) Transformation of 3D displacement matrix from time domain to frequency spectrum; (**b**) excitation signal in time domain and frequency spectrum.

For the analysis, the excitation signal from the analog output was adjusted to a time-domain waveform based on signal processing theory. Subsequently, it was transformed into a frequency spectrum (Figure 10b) by using the fast Fourier transform (FFT) (Figure 10a).

A three-dimensional displacement matrix of the oscillating steel plate in the *Z*-axis direction, where the third dimension of the matrix represents time, was then used to determine the modal shapes. To describe each dimension of the matrix, a representation model was created, shown in Figure 10a. The product of the number of rows and columns gives the number of points (facets) at which displacements were measured. Each of these points has a specific characteristic function dependent on time t. These displacement functions of all points were transformed from the time domain to the frequency spectrum by a fast Fourier transform (FFT) (Figure 10a) [28,33,34,35,36,37,38,39,40,41,42,43,44,45,46,47,48,49,50,51,52,53,54,55].

**Figure 11:**
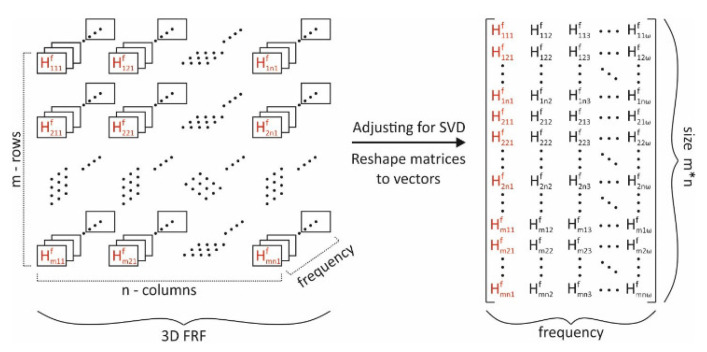
Transformation of the FRF matrix into vector notation.

Subsequently, the FRF matrix was determined based on the response in the form of the frequency spectrum of the displacement matrix, which is influenced by the frequency spectrum of the excitation signal, and transformed into a vector notation (Figure 11).

**Figure 12:**
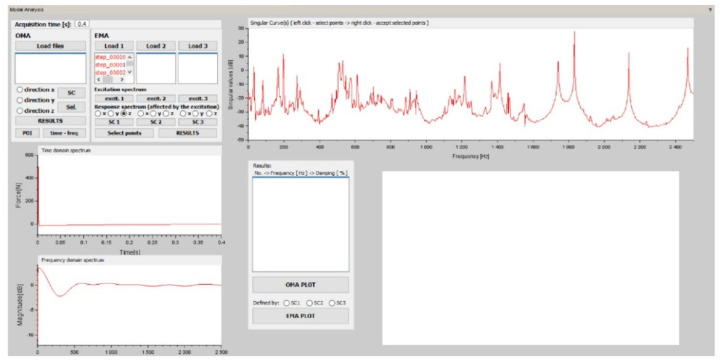
Singular curve.

By plotting the singular values on the frequency axis, a singular waveform was obtained whose peaks indicated the presence of modes of oscillation (Figure 12). The respective frequencies of the individual peaks are the natural or resonant frequencies. For each natural frequency, there is an intrinsic mode of oscillation. The eigenmodes of oscillation are exported for the selected frequency from the FRF matrix.

Another modal parameter that needs to be determined is the damping coefficient of the mode of oscillation. Since the Scilab software does not provide the ability to automatically select the half-power points based on the peaks, the nearby frequencies need to be manually selected.

**Figure 13:**
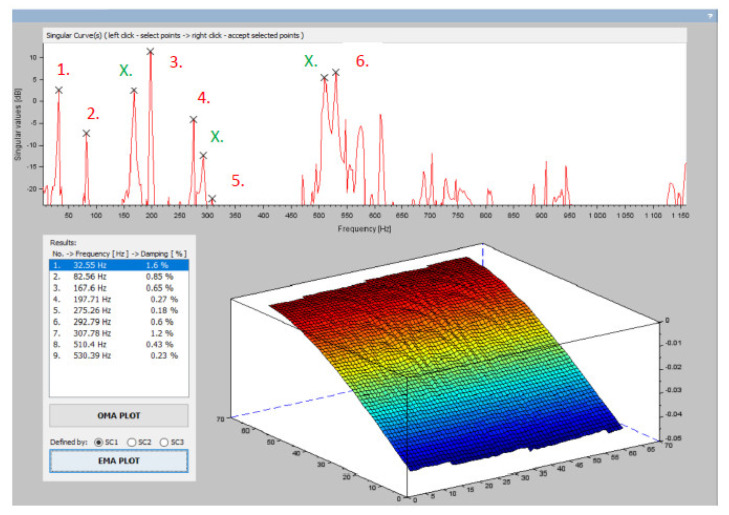
Resulting modal parameters obtained with the application module.

Figure 13 shows the natural frequencies and the corresponding mode damping coefficients determined from the *Z*-axis displacement matrix obtained by the DIC method (points 1–6). It is also possible to see peaks in the singular waveform that are not natural frequencies of the oscillating steel airfoil, but represent modes resulting from the fit (points X.). In the same way, the modal parameters in the X and Y axis directions can be analyzed in the proposed application [28].

**Figure 14:**
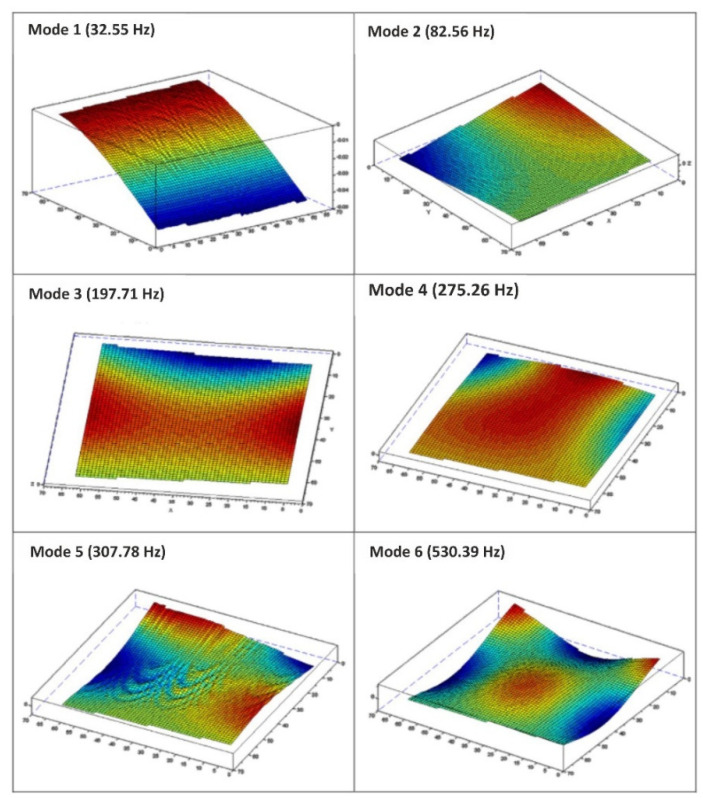
Modal shapes and corresponding EMA eigenfrequencies.

Each natural frequency has its own specific mode of oscillation. The individual mode shapes with their respective frequencies are shown in Figure 14.

## 5. FEM Modal Analysis

The correctness of the results obtained by experimental modal analysis based on DIC measurements was verified numerically. There are many numerical methods such as peridynamics [56], discrete element method [57], damage mechanics [58], and others, but due to the availability of software, we used the finite element method (FEM) to verify our solution. The numerical model was constructed in the form of a shell element to which the material properties of the steel and the dimensions of the real measured steel plate were assigned. The boundary condition of the fit was simulated by taking all degrees of freedom of motion at the bottom edge of the numerical model. The mesh was meshed from quadrilateral elements.

**Figure 15:**
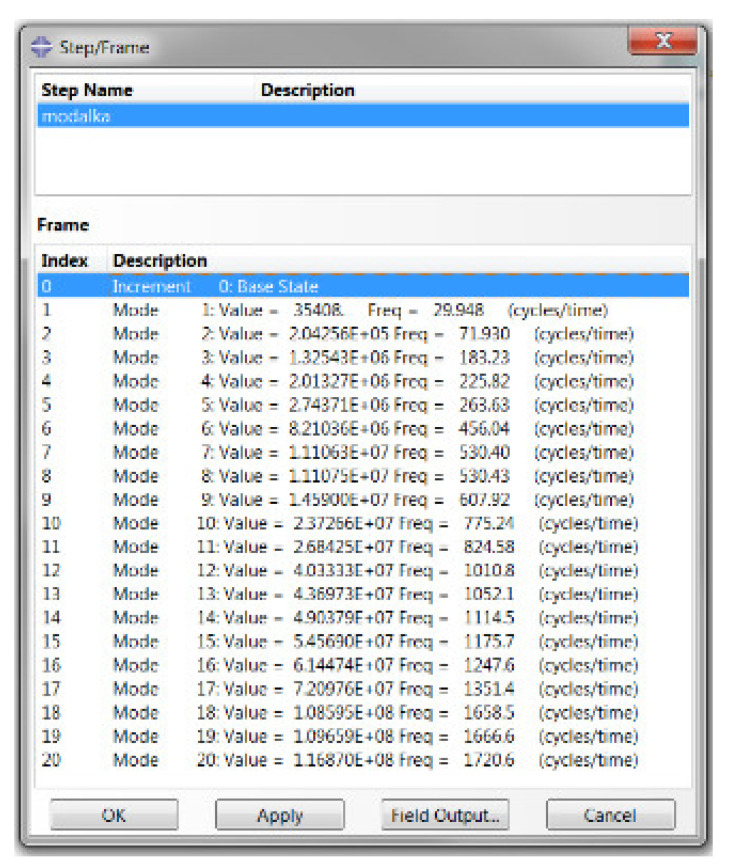
Eigenfrequencies of the modes determined by the FEM method.

**Figure 16:**
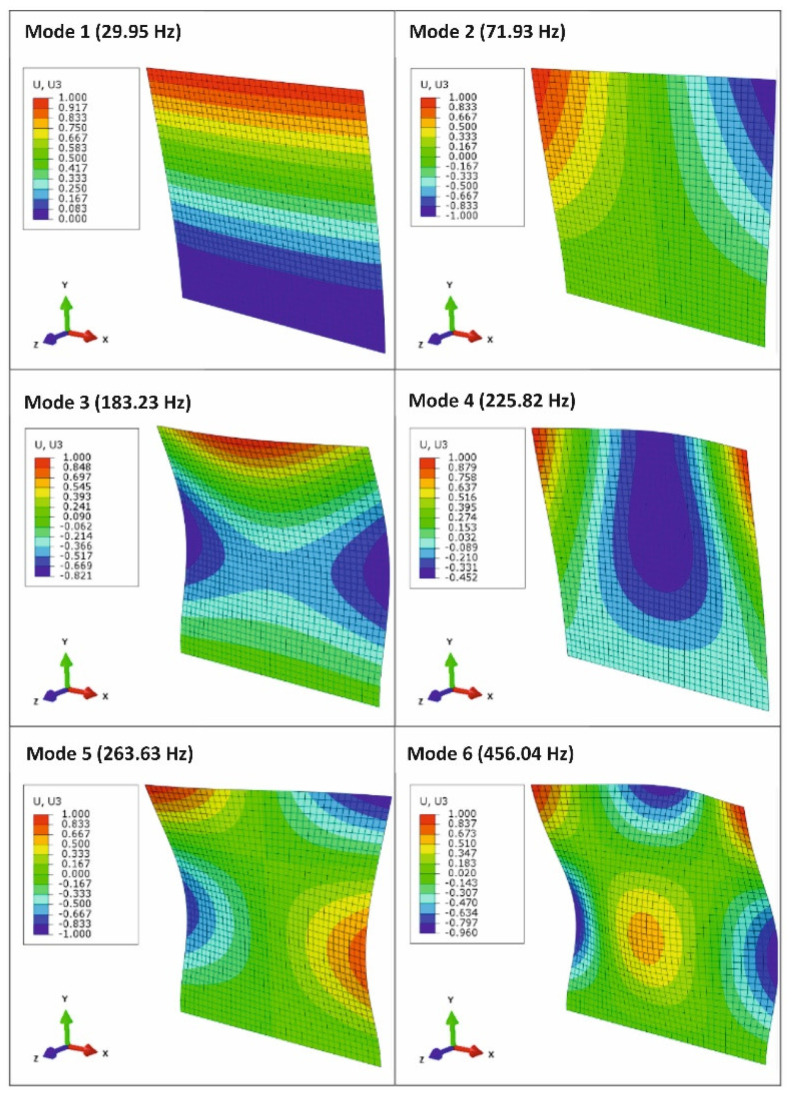
Modal shapes (Z direction) and corresponding natural frequencies obtained by the FEM method.

The resulting frequencies obtained by the FEM method are shown in Figure 15. To compare the results of the natural frequencies and modal shapes, the first six modes were selected whose natural shapes are shown in Figure 16 [28].

## 6. Discussion

The resulting eigenfrequency values obtained experimentally by modal analysis using the DIC method and numerically by the FEM method are shown in Table 1. The differences of the natural frequencies in Hertz and percentage are also given in Table 1.

**Figure 17:**
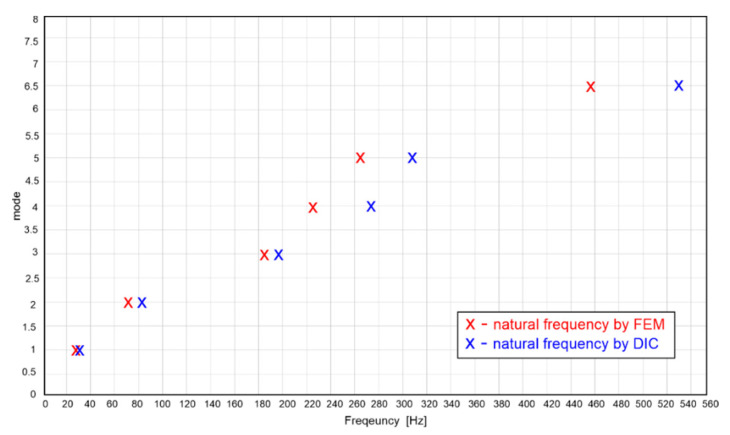
Comparison of eigenfrequency results obtained by EMA analysis and FEM analysis.

In Figure 17, the deviations of the resulting natural frequencies of the different modes of oscillation obtained by numerical and experimental analysis are plotted [28].

The differences arising in the results are attributed to the choice of boundary conditions in the numerical FEM method. In the experiment, it is difficult to achieve a perfect fit of the analyzed sample.

## 7. Conclusions

Currently, either of the numerical modal analyses are used to estimate the modal parameters, or if a more accurate estimation corresponding to the real material properties and boundary conditions of the fit is required, an experimental solution is approached. The commonly used experimental modal analysis procedures are currently conditioned by the application of acceleration sensors on the object under analysis, by means of which the resulting frequency response function is obtained.

This paper describes a new approach to estimate the natural (resonant) frequencies, as well as the corresponding modal oscillation shapes and damping coefficients, based on the results of measurements obtained by the optical digital image correlation method. The resulting modal shapes are obtained based on a displacement matrix.

The main advantage of this new methodology is the fact that, by using high-speed ka-measurements, a full-field analysis of the imaged area of the object is possible, which implies that responses at all points of the object can be obtained on the basis of a single measurement. Conventional methods, with their acceleration sensors, can negatively affect the modal parameters of the system by their mass for sensitive analyzed objects. The full-field analysis by the DIC method makes it possible to determine the responses of any point on the analyzed object without the additional mass of the sensors.

The application module which has been designed in Scilab is able to determine the modal parameters based on the measured displacement matrix by the DIC method. The aforementioned application is able to determine natural frequencies, modal shapes, and damping coefficients based on experimental modal analysis when the FRF function takes into account the excitation of the system. It is also possible to estimate the modal parameters based on the principle of operational modal analysis when the excitation of the system is unknown.

## Figures and Tables

**Table 1:** Resulting eigenfrequency values obtained by EMA analysis and FEM analysis.

Mode	EMA Analysis [Hz]	FEM Analysis [Hz]	Difference [Hz]	Difference [%]
Mode 1	32.55	29.95	2.6	7.99
Mode 2	82.56	71.93	10.63	12.88
Mode 3	197.71	183.23	14.48	7.32
Mode 4	275.26	225.82	49.44	17.96
Mode 5	307.78	263.63	44.15	14.34
Mode 6	530.39	456.04	74.35	14.01

## Data Availability

Not applicable.
